# Laparoscopic left hepatectomy for a patient with an absence of portal bifurcation using real-time imaging: a case report

**DOI:** 10.1186/s40792-024-01945-3

**Published:** 2024-06-11

**Authors:** Shugo Mizuno, Yusuke Iizawa, Akihiro Tanemura, Benson Kaluba, Daisuke Noguchi, Takahiro Ito, Aoi Hayasaki, Takehiro Fujii, Yasuhiro Murata, Naohisa Kuriyama, Masashi Kishiwada

**Affiliations:** Department of Hepatobiliary Pancreatic and Transplant Surgery, Mie University School of Medicine, 2-174 Edobashi, Tsu, Mie 514-8507 Japan

**Keywords:** Absence of portal vein bifurcation, Portal vein anomaly, Laparoscopic left hepatectomy, ICG fluorescence real-time imaging

## Abstract

**Background:**

Absence of portal bifurcation is an extremely rare anomaly that should be recognized preoperatively, especially prior to a major hepatectomy.

**Case presentation:**

A 45-year-old woman presented with abdominal pain, and abdominal computed tomography (CT) revealed dilatation of both the common bile duct (CBD) and intrahepatic bile duct (IHBD). Endoscopic retrograde cholangiopancreatography (ERCP) showed CBD and IHBD stones (B2 and B4). The CBD stones were removed, but the IHBD stones could not be, yet there was no evidence of malignancy at the site of IHBD stenosis. Enhanced CT revealed a dilated IHBD, while three-dimensional CT images showed the left portal vein running through the ventral side of the middle hepatic vein, which was diagnosed as the absence of portal vein bifurcation (APB). Laparoscopic left hepatectomy was successfully performed using real-time indocyanine green (ICG) fluorescence imaging.

**Conclusion:**

Surgeons should be aware of the possibility of APB, a rare portal vein anomaly, before performing major hepatectomy. Real-time ICG fluorescence imaging may be helpful to ensure the precise anatomy of the liver during laparoscopic surgery.

## Introduction

The absence of portal vein (PV) bifurcation (APB) is an extremely rare anomaly that has been reported in 0.03–2% of all cases in previous anatomical studies [1]. For patients requiring liver transplantation, hepatectomy, or PV embolization, this anomaly should be recognized before treatment for patient safety [2]. Although surgical methods for hepatectomy in several APB cases have previously been reported [3–7], patients in all these cases underwent the open method, as it is a very complicated operation. To the best of our knowledge, this is the first report of laparoscopic left hepatectomy in a patient diagnosed with APB in whom a single intrahepatic PV was observed running from the right anterior section to the left lateral section of the liver.

## Case presentation

**Fig. 1:**
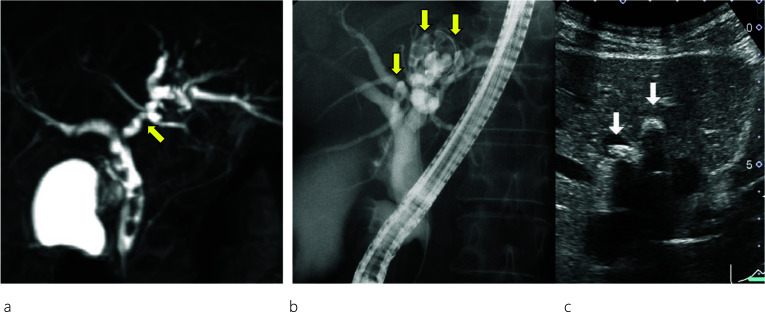
Image findings. Magnetic resonance cholangiopancreatography (MRCP) (**a**) shows common bile duct (CBD) stones and stenosis at the confluence of B2 and B3. Endoscopic retrograde cholangiopancreatography (ERCP) (**b**) reveals intrahepatic bile duct (IHBD) stones in B2 and B4 (yellow arrows). Abdominal ultrasound scan (**c**) shows IHBD stones in B2 and B4 (white arrows)

**Fig. 2:**
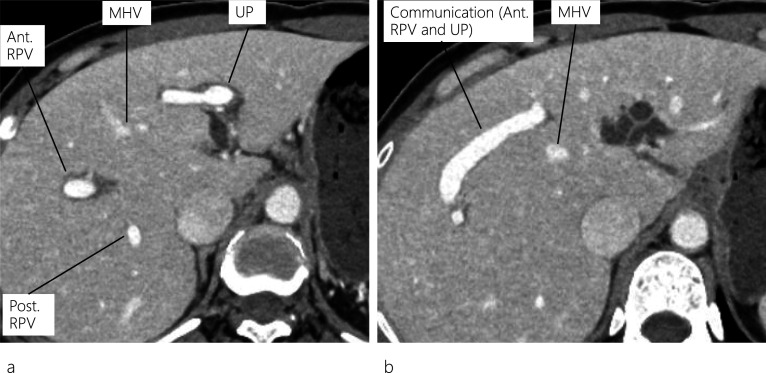
Enhanced CT images. Enhanced CT scan reveals a dilated IHBD in the left lobe and a communication between the right portal vein and umbilical portion (**a**, **b**). CT: computed tomography; IHBD: intrahepatic bile duct; MHV: middle hepatic vein; Ant. RPV: anterior right portal vein; Post. RPV: posterior right portal vein, UP: umbilical portion

**Fig. 3:**
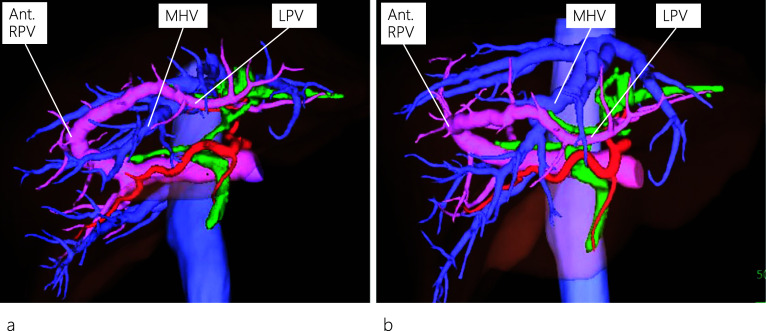
Three-dimensional reconstruction of the enhanced CT images. The left hepatic artery and bile duct are separated from the left portal vein (LPV) instead of being located in the left Glisson’s sheath as viewed from a left anterior oblique position (**a**). LPV, viewed from a neutral position, runs through the ventral side of the middle hepatic vein (MHV) (**b**). MHV: middle hepatic vein; Ant. RPV: anterior right portal vein; LPV: left portal vein

A 45-year-old female patient visited a local hospital with abdominal pain. Blood tests revealed elevated liver enzyme levels, and abdominal computed tomography (CT) revealed dilatation of both the common bile duct (CBD) and intrahepatic bile duct (IHBD). The patient underwent magnetic resonance cholangiopancreatography (MRCP) and endoscopic retrograde cholangiopancreatography (ERCP). MRCP showed CBD stones and stenosis at the confluence of B2 and B3 (Fig. 1a), while ERCP revealed IHBD stones in B2 and B4 (Fig. 1b), which were also observed on abdominal ultrasound (US) (Fig. 1c). Brushing cytology and biopsy at the site of the IHBD stenosis were negative for malignancy. Although the CBD stones were removed, the IHBD stones could not be; therefore, she was referred to our hospital for surgical treatment. Further examination could not be performed around the site of the IHBD stenosis because of impacted stones. Therefore, laparoscopic left hepatectomy was scheduled for suspected intrahepatic cholangiocarcinoma resulting from the impacted stones. However, to properly delineate the anatomy of the liver, enhanced CT was performed, which revealed a dilated IHBD in the left lobe and communication between the right PV and the umbilical portion (Fig. 2a, b). Based on the enhanced CT data, three-dimensional (3D) CT images were reconstructed, which revealed that instead of being located in the left Glisson's sheath, both the left hepatic artery and bile duct were separated from the left PV (LPV) (Fig. 3a). They also revealed an LPV running through the ventral side of the middle hepatic vein (MHV) (Fig. 3b). Based on these findings, the patient was diagnosed with APB and scheduled to undergo laparoscopic left hepatectomy.

**Fig. 4:**
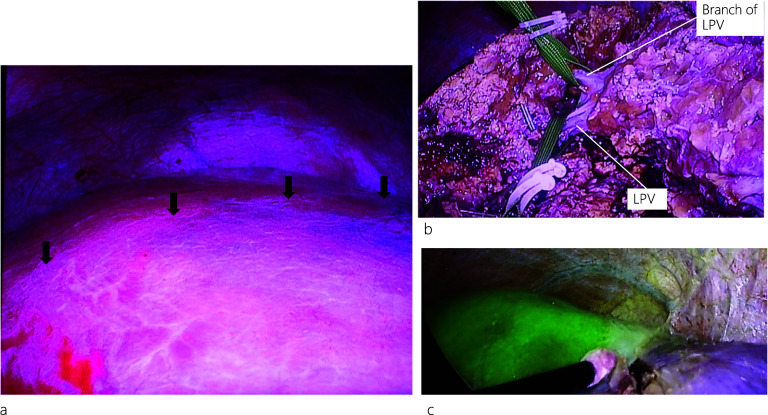
Intraoperative findings during laparoscopic left hepatectomy. A demarcation line (black arrows) appears after injecting indigo carmine (10 mg) into the LPV above the MHV under intraoperative ultrasound (US) guidance (**a**). The LPV and its branch are encircled (**b**). After clamping the LPV and its branch, negative staining using indocyanine green (ICG) fluorescence real-time imaging shows hepatic perfusion and a clear demarcation line (**c**)

**Fig. 5:**
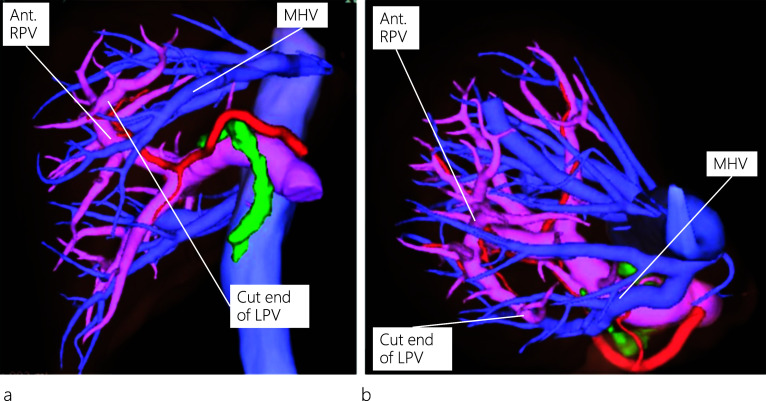
Three-dimensional reconstruction of postoperative enhanced CT images. Both the MHV and Ant. RPV are preserved, as viewed from neutral (**a**) and left posterior oblique (**b**) positions. CT: computed tomography; MHV: middle hepatic vein; Ant. RPV: anterior right portal vein; LPV: left portal vein

During the operation, when we first encircled and clamped the left Glisson’s sheath, no demarcation lines appeared. Under intraoperative US guidance, indigo carmine (10 mg) was injected into the LPV above the MHV, and a temporary demarcation line appeared (Fig. 4a). Subsequently, the hepatic parenchyma was transected along the demarcation line and encircled the LPV. After clamping the LPV and injecting indocyanine green (ICG: 2.5 mg) through a peripheral vein, negative staining using ICG fluorescence real-time imaging showed hepatic perfusion, and the demarcation line was clearly visible (Fig. 4b, c). Transection of the liver parenchyma was continued, and left hepatectomy was completed. Intraoperative frozen section histopathology of the cut end of the left hepatic bile duct was negative for malignancy. The operation time was 413 min, and blood loss was 83 mL. Pathological reports showed only inflammation of the bile ducts, without any malignancy (Fig. 5a–c), while postoperative 3D CT images showed preserved MHV and right PV. The patient developed an intraabdominal abscess, for which CT-guided drainage was performed on postoperative day (POD) 13. After successful treatment, the drain was removed, and the patient was discharged on POD 28.

## Discussion

Anomalies of the PV branches at the hepatic hilum are not so common compared with those of the hepatic arteries, hepatic veins, and biliary ducts [8, 9]. APB is a rare anomaly, first reported by Couinaud [10, 11]. The adult PV develops from the vitelline (future main PV) and umbilical veins (future vertical segment of the left PV) during the 4th to 10th weeks of gestation. The absence of the horizontal part of the LPV or APB can be due to failure of the anastomosis between the caudal portion of the right vitelline and left umbilical veins [1, 2].

Regarding the anatomy of the bile ducts in patients with APB, Terasaki et al. [12] reported two patients with different bile duct characteristics, in whom the left hepatic duct ran either inside the liver along the PV or separately down into the common hepatic duct. The differences in running patterns between the PV and the bile duct can be explained from an embryological perspective. In our patient, an ordinary left bile duct was present, and IHBD stones were identified in the dilated B2 and B4. It is noteworthy that on routine preoperative plain CT images, the APB anomaly was not detected owing to the limitation of the images in outlining the detailed anatomy of the liver vasculature. However, the anomaly was detected on the preoperative enhanced CT images, which allowed us to reconstruct 3D images and view the detailed anatomy of the liver vasculature from different positions (neutral and left anterior oblique positions), as shown in Fig. 3.

Focusing on hemihepatectomy (right or left hepatectomy), this anomaly should be recognized before surgery to assess and determine the PV blood flow. Until now, there have been several reports of right hepatectomy by preserving the main and left PVs in patients with APB [5, 6, 13]. Among them, two patients experienced PV thrombosis after surgery, and one patient died of liver failure [5, 13]. When considering surgical treatment for patients with APB, surgeons must examine the precise arterial, biliary, and PV anatomy and consider the risks associated with right hepatectomy preoperatively. In contrast, left hepatectomy can be safely performed because surgeons do not have to identify the left PV at the perihilar area, and the right PV can easily be preserved.

It is difficult to determine the demarcation line when performing left hepatectomy in patients with APB. In this situation, the only option is to split the liver parenchyma along the Rex–Cantlie line and separate the PV from the anterior section. Besides our limited experience with this technique, we initially attempted PV puncture by injecting indigo carmine (10 mg) into the LPV above the MHV under US guidance to visualize the demarcation line. However, only a temporary demarcation line appeared (Fig. 4a). The hepatic parenchyma was transected along this temporary demarcation line, and the LPV was dissected and encircled. We then injected ICG (2.5 mg) into the peripheral vein, and after clamping the LPV, we could precisely see the well-established demarcation line (Fig. 4c) using ICG fluorescence real-time imaging and completed the left hepatectomy. However, owing to the complexity of liver resection, the hepatic cut surface was not smooth. This could have promoted the formation of abscesses at the cut margins and eventually lead to a postoperative intraabdominal abscess, for which the patient was successfully treated with CT-guided drainage and antibiotics.

ICG fluorescence imaging has been used for intraoperative identification of hepatic tumors and segmental boundaries during laparoscopic hepatectomy, particularly for anatomical resection [14, 15]. Recently, this technique has become common during laparoscopic hepatectomies because fluorescence images can allow surgeons to identify the segmental boundaries of the hepatic parenchyma with or without blood perfusion and thus guide surgeons to complete anatomic resections laparoscopically [16]. Although the histopathological report revealed this case as a benign disease, ICG fluorescence real-time imaging was used preoperatively, and we planned to perform a laparoscopic anatomical liver resection based on a strong suspicion of intrahepatic cholangiocarcinoma due to the impacted IHBD stones. Therefore, this real-time intraoperative imaging technique might be useful for patients with APB requiring hepatectomy by facilitating the visualization of the demarcation line and consequently aiding in liver parenchyma resection. Although the assessment of hepatic perfusion by fluorescence imaging following the intravenous injection of ICG cannot be used repeatedly, this modality can allow surgeons to perform safe and accurate laparoscopic hepatectomies.

## Conclusions

Here, we present the case of a patient diagnosed with APB who underwent laparoscopic left hepatectomy. Before performing major hepatectomies, surgeons should be aware not only of the occurrence of this rare PV anomaly, but also of hepatic arterial and biliary anomalies. To ensure the precise anatomy of the liver during hepatectomy, ICG fluorescence real-time imaging may be helpful.

## Data Availability

All data supporting the conclusions of this article are included within the published article and the accompanying images.

## Abbreviations

CT: Computer tomography
CBD: Common bile
IHBD: Intrahepatic bile ducts
ERCP: Endoscopic retrograde cholangiopancreatography
MHV: Middle hepatic vein
APB: Absence of portal vein bifurcation
ICG: Indocyanine green
MRCP: Magnetic resonance cholangiopancreatography
3D: Three-dimensional
PV: Portal vein
LPV: Left portal vein
MHV: Middle hepatic vein
US: Ultrasonography
POD: Post-operative day

## References

[1] Marks C (1969). Developmental basis of the portal venous system. Am J Surg.

[2] Chaib E (2009). Absence of bifurcation of the portal vein. Surg Radiol Anat.

[3] Fraser-Hill MA, Atri M, Bret PM (1990). Intrahepatic portal venous system: variations demonstrated with duplex and color Doppler US. Radiology.

[4] Hardy KJ, Jones RM (1997). Failure of the portal vein to bifurcate. Surgery.

[5] Teraoku H, Arakawa Y, Yoshikawa M (2016). Complication of portal vein thrombosis after right hemihepatectomy in a patient lacking the portal vein bifurcation. J Med Invest.

[6] Spampinato MG, Baldazzi G, Polacco M (2012). Right hemihepatectomy in presence of congenital absence of portal vein bifurcation: a challenging but feasible procedure. J Am Coll Surg.

[7] Yamamoto H, Nagino M, Kawabata Y (2000). Resection of a hilar cholangiocarcinoma in a patient with absent portal bifurcation. Hepatogastroenterology.

[8] Varotti G, Gondolesi GE, Goldman J, Wayne M, Florman SS, Schwartz ME, Miller CM, Sukru E (2004). Anatomic variations in right liver living donors. J Am Coll Surg.

[9] Kamel IR, Kruskal JB, Pomfret EA, Keogan MT, Warmbrand G, Raptopoulos V (2001). Impact of multidetector CT on donor selection and surgical planning before living adult right lobe liver transplantation. AJR Am J Roentgenol.

[10] Couinaud C (1953). Etude de la veine porte intrahepatique. Presse Med Paris.

[11] Couinaud C. Surgical anatomy of the liver revisted Igaku-shoin, Tokyo. 1996. p. 22–5.

[12] Terasaki F, Yamamoto Y, Sugiura T, Okamura Y, Ito T, Ashida R, Ohgi K, Aramaki T, Uesaka K (2021). Description of the vascular anatomy of livers with absence of the portal bifurcation. World J Surg.

[13] Koh MK, Ahmad H, Watanapa P (1994). Beware the anomalous portal vein. HPB Surg.

[14] Ishizawa T, Zuker NB, Kokudo N, Gayet B (2012). Positive and negative staining of hepatic segments by use of fluorescent imaging techniques during laparoscopic hepatectomy. Arch Surg.

[15] Ueno M, Hayami S, Sonomura T (2018). Indocyanine green fluorescence imaging techniques and interventional radiology during laparoscopic anatomical liver resection (with video). Surg Endosc.

[16] Terasawa M, Ishizawa T, Mise Y, Inoue Y, Ito H, Takahashi Y, Saiura A (2017). Applications of fusion-fluorescence imaging using indocyanine green in laparoscopic hepatectomy. Surg Endosc.

